# Treatment of Acute Otitis Media1Read at the March meeting of the Erie County Medical Association.

**Published:** 1905-06

**Authors:** W. Scott Renner

**Affiliations:** Buffalo, N. Y.


### Treatment of Acute Otitis Media.[1]

By W. SCOTT RENNER, M. D., Buffalo, N. Y.

TWO things are essential to the proper treatment of any diseased organ: first, examination of the organ to determine the indications for treatment; second, treatment of the diseased organ according to the indications discovered by the examination. No practitioner would think of treating a suspected pneumonia without percussion and auscultation of the chest; but many able physicians will treat an acute middle ear trouble without once examining the tympanic membrane, the auditory canal, the mastoid process or the upper respiratory tract. Yet, a direct inspection of these parts and that daily, is as essential to the proper treatment of an otitis media, as it is to auscultate the chest in pneumonia.

In olden times, otitis media was not treated; at present, it is cared for but often badly treated. Dirty dressings of the auditory canal with soothing ointments, warm oils or laudanum are useless. More progressive men prescribe profuse injections of boracic acid and other solutions, or installations of carbolic acid and glycerine, which are perfectly proper if they are discontinued when other steps become indicated. Some general practitioners will tell us that we are over anxious because we see only the severe cases. They state that discharging ears are only insignificant troubles, especially in children, which cure themselves.

1. Read at the March meeting of the Erie County Medical Association.

They are, however, deceived by an overstock of faith when they think that an otitis is well cured because the ear shows no evidence of pain or discharge. It may be full of danger like a quiescent appendicitis. It may insidiously continue its march to a brain abscess, meningitis, or sinus thrombosis, or to deafness, which cripples the usefulness of the patient.

Otology today does not deserve the reproach which was due it twenty years ago. At present, instead of the use of profuse injections and insufflations into, and bacteriocidal dressings of the auditory canal, which is not the diseased organ in this case, we treat, as far as possible, the middle ear according to modern surgical methods, and the otologist is enabled to know just what procedure to carry out by the indications which inspection of the auditory canal and palpation of the mastoid process give him.

Acute processes of the middle ear are classified variously by different authors and, therefore, it becomes necessary to precede our description of their treatment by a concise definition of the morbid processes included under the term, acute otitis media. Many authors describe three principal subdivisions: first, middle ear catarrh; second, simple acute otitis media or simple acute inflammation of the middle ear; and third, acute purulent inflammation of the middle ear. These three cannot always be sharply distinguished in their early stages, and the first may, at any time, be changed into one of the last two. These two are the same condition, one of which goes on to suppuration, the milder form not reaching that state, either from the lack of virulence or as a result of treatment.

Acute middle ear catarrh is, according to these authors, not the result of infection of the middle ear, but the changes are brought about in the middle ear when the Eustachian tube is obstructed. Such obstruction prevents the normal change of air between the middle ear and the pharynx; thus the air in the middle ear becomes rarified. This results in hyperemia and perhaps transudation of serum from the side walls of the tympanic cavity. These cases are not catarrh in the true sense of the word and there is not always an exudate into the tympanum. They occur usually in obstructive cases of the nose and nasopharynx. Children with adenoid vegetations often suffer from this form of catarrh. It is these children that have frequent attacks of earache which sometimes end in a suppurative otitis media. The tympanic membrane presents, on inspection, a characteristic appearance. The membrane is drawn in, causing a more or less marked formation of a posterior fold of the membrane and sometimes the formation of an anterior fold with a slight redness of the membrane. This redness of the tympanic membrane is not the result of inflammation, but is due to the hyperemic condition of the mucous membrane within the tympanic cavity which shines through the tympanic membrane. The membrane does not appear swollen and the light reflex is not at all or very little changed. The region of the handle of the hammer is not changed, though perhaps its bloodvessels may be more filled than normal. A later result of this negative pressure, as I said before, is the transudation of serum into the tympanic cavity.

This so-called catarrhal condition has been described to prevent confusing it with the two inflammatory forms which are the same condition, except that the more severe one goes on to the formation of pus in the middle ear. They are caused by a bacteriological infection of the middle ear in the course of an acute cold, scarlet fever, measles, typhoid, diphtheria, and the like, while the exudate found in the middle ear is an inflammatory product and not transudate. This exudate is serous, mucus, or purulent according to the severity and stage of the inflammation. This inflammatory disease is recognised from the appearance of the membrane. When seen early, the lower part of the membrane may still retain its sheen and the light reflex will still be found, but the region of the handle of the hammer becomes swollen. The short process is not so evident; it may be just distinguishable, or not at all to be seen. There may be in such a case only a small amount of serous exudate which may become absorbed. The purulent form commences in this manner also, but the signs apparent on the drum membrane increase in severity until the whole membrane is red, swollen, and bulging.

Now, I do not think any doubt can exist as to what class of cases we should include under the name of acute middle ear inflammation and we may proceed to a consideration of its treatment. It is not a trivial disease, therefore rest in bed should be enjoined; this is especially necessary if there is severe pain, fever and signs of severe inflammation in the tympanum. The patient should be kept upon a low diet and all stimulants should be avoided. Calomel, followed by a saline should be administered. Even in the mildest cases, the patient should, if possible, be confined to his room. Until signs of accumulation of exudate are manifest by a bulging tympanic membrane, applications of heat should be made over the ear and mastoid process. The external auditory canal may also be irrigated every hour with a warm sterile lotion. This irrigation is usually quite soothing, but should be done very gently to avoid injury to the drum. I am, at times, inclined to discontinue its use, because it has a tendency to macerate the canal and cause desquamation which disguises more or less the tympanic picture. The external application of heat is often also soothing, but probably has very little to do with the reduction of the inflammatory condition. It may be made by means of dry heat, such as the hot water bag, or moist applications may be applied for two hours at a time and then allowing two hours to intervene. Such applications may be kept hot in a chafing dish or hot water bath.

A very useful solution for this purpose employed frequently in the German clinics, is liquor aluminum acetate; about four drams of this can be used in a half pint of water, kept warm in a chafing dish and changed every few minutes by the patient. In many individuals, especially those who are corpulent, hot applications have a tendency to increase pain; in such patients ice may be substituted for heat. There are many things recommended in these cases which are either useless or interfere with our observation of the progress of the inflammation. In the past, leeches have been extensively used, either in front of the tragus or over the mastoid process. Their application in our antiseptic times seems to be wrong. They also cause a light inflammatory reaction in the neighborhood of the bite, which makes it difficult for one to say whether the tenderness on pressure is due to the leech bite inflammation, or to the inflammation of the underlying periosteum and bone. For similar reasons, fly blisters, tincture of iodine, gray salve and other similar things should not be applied over the mastoid; they simply disguise the condition of the part.

I have already said that sterile solutions may be used in the auditory canal, but doubt whether it is advisable to use them or not; they, of course, prepare the canal by making it aseptic for an incision of the drum, should it become necessary. Most fluids which are poured into the ear are positively harmful, or at least, absolutely useless; among these are laudanum, warm sweet oil, goose grease, bear grease, and the like. I recently had some trouble in removing from an auditory canal a large amount of vaseline which had been poured into the ear while warm. Ten per cent. carbolised glycerine is used in some places quite extensively to abort these cases. It also may be said to be useful as an antiseptic to the canal; it sometimes, however, acts as a slight cauterant and the slough left only serves to confuse the examiner.

Should this treatment fail to relieve the pain and should the fever and inflammation of the drum continue for more than twenty-four hours, or should the symptoms increase and the tympanic membrane show signs of bulging, a paracentesis should be made. This incision should be made only after the auditory canal is thoroughly cleansed with an antiseptic solution, and dried so that a satisfactory view of the tympanic membrane may be had.

The hands and instruments of the operator should be made sterile, as they would be in a major operation, and this at each dressing of the case. The incision through the drum in most cases should be made in the posterior inferior quadrant, a short distance from its outer border and parallel with it. This position of the incision insures good drainage; first, from its position in the lower part of the membrane; and, again, because it is made at right angles to the radiating fibers of the membrane. The incision should not be a simple puncture of the membrane, but it should be as long as it possibly can be made within the arc of the posterior inferior quadrant.

After the tympanic membrane has been incised, the external auditory canal should be dried, that is, all pus, blood, or serum should be removed and a narrow strip of sterilised nonmedicated gauze should be placed loosely in the auditory canal, the inner end of which should be placed as near the incision as possible. Care should be taken not to pack this dressing in the canal, as this would interfere with the object of it; unfortunately the dressing cannot be carried through the incision into the tympanic cavity. Were that possible, paracentesis of the drum would be an ideal surgical operation. Some recommend placing an external dressing of absorbent cotton over this dressing with a bandage, leaving it for twenty-four hours. This will do in cases where the discharge is small in amount. The best procedure, in my experience, is to change this dressing as often as it becomes soaked with secretion. I do not make a practice of washing out these cases after the incision, unless there is considerable desquamation of epithelium which cannot be removed with absorbent cotton.

The physician should see these patients at least once daily, should thoroughly dry the canal and if necessary irrigate it gently to remove the desquamating epithelium. Under no other circumstances would I recommend the irrigation of the canal. After this, the condition of the membrane, the appearance of the posterior superior wall of the canal and the condition of the mastoid as to pain, tenderness and swelling, should be made out. This plan of treatment should be carried out until the patient is well or until the condition of the mastoid makes operative interference necessary. The practice of washing out the auditory canal to heal up a middle ear discharge is, per se, nonsense. The diseased part is beyond the tympanic membrane and can but rarely be reached by the solution; and, besides, washing out abscess cavities is, at the present time, considered poor surgery. For a similar reason, washing out the tympanic cavity through the Eustachian tube is not a surgical procedure and now is scarcely ever recommended.

When a purulent otitis media comes under my observation, after perforation of the drum has taken place from over distention, I clean the auditory canal the same as I did at the daily dressings after incising the drum, and I also inspect it in the same manner; if the perforation in the drum does not seem large enough for free discharge of pus or if there are already signs of mastoid retention, I do a paracentesis in the same manner as in the unperforated cases. In making the incision in these cases, I try to start if possible from the rupture; that is, if it is favorably located. The daily treatment is the same in these cases. Formerly, the air douche by Politzer's method, or with the Eustachian catheter was used daily. Some authors are still in favor of its use; others condemn it, stating that it irritates the parts when they need rest, and drives the secretion into the mastoid antrum; as it is already there, however, this can do very little harm. It seems to me, though, to have no particular advantage; yet, it must be used in the so-called catarrhal cases and in acute inflammatory cases after the discharge has stopped and the perforation healed, but then very gently, for there is danger of reopening the perforation.

The question is often asked, "Should the tonsils or adenoids be removed during an acute otitis media?" I should say, certainly not; except in the long drawn out cases, where the treatment is practically the same as that of chronic otorrhea. In many cases, the discharge seems to come through the Eustachian tube into the tympanic cavity.

### INDICATIONS FOR THE MASTOID OPERATION.

In all cases of purulent otitis media, the mastoid antrum is likewise involved, but clinical experience shows us that a timely and large paracentesis of the drum will usually drain the antrum as well as the tympanum, and that it is cured in this way more quickly than a mastoid wound could heal. We also know that mastoid symptoms that persist after a paracentesis are not produced by involvement of the mastoid antrum, but by retention of pus in the mastoid cells and in inflammation of their walls. These cells are situated in different parts of the temporal bone. Therefore, when a good free paracentesis does not cause the disquieting symptoms to disappear; that is, if constant pain and tenderness persist in the region of the mastoid; if sagging of the posterior wall of the auditory canal is present; and if pus appears in abundance in the auditory canal after its removal, it is then time to follow up the incision of the tympanum, by an opening and an exenteration of the mastoid cells until all diseased parts are thoroughly removed and the antrum freely exposed. If the above named symptoms accompanied by some fever persist twenty-four hours after a free incision of the drum, the operation should be done, it seems to me, at once; it is useless to attempt to cure the mastoid trouble by application of ice over the mastoid and frequent warm irrigation of the Eustachian tube; such delay is dangerous, for we cannot tell by the external symptoms how extensive the internal changes may be.

If the patient comes to us with a periosteal mastoid abscess, even if the temperature is not a hundred, it is imperative that the operation should be done as quickly as it is in the power of the surgeon to arrange to do it. These are neglected cases with destruction and fistula through the mastoid cortex. Paracentesis in such cases, or an enlargement of the size of the perforation in the tympanic membrane can do no good. They are accompanied usually, by an extradural abscess, and delay is absolutely very dangerous. It is necessary in doing the mastoid operation to always open the mastoid antrum and every mastoid cell, if we wish to avoid future complications. The more radical the operation, the better for the patient. The lesions found in doing the mastoid operation are usually more extensive than suspected, hence all diseased bone should be removed down to the dura and every bolster of granulation on the dura should be exposed in every direction, until healthy dura can be seen on every side of the pad of granulation tissue and should fistulas lead through the dura into the brain, they should be followed to their source, and should signs of sinus thrombosis exist, the thromboses should be removed and if necessary the jugular vein be ligated and cleaned out. Early operations upon the mastoid do not usually reveal more than an extradural abscess, which if properly treated, is a harmless intracranial complication.

The mastoid operation is the ideal surgical way of treating an otitis media and if it were not for the waste of time involved, it would always be the best way of insuring a complete cure of the discharge, leaving an intact healthy tympanic membrane, for the gauze drainage can always be placed directly in the cavity from which the pus comes, which cannot be done where drainage is attained through an incised tympanic membrane. When a paracentesis does not result in a reasonable time in a complete cure of aural discharge, the mastoid antrum should be opened to prevent the life long risks which result from neglected bone disease and deafness. The treatment of the neglected cases, that is, chronic otorrheas, and the like, constitutes another chapter of otology, the consideration of which is foreign to the object of the present paper.

361 Pearl Street.

